# Annotations

**Published:** 1902-10-18

**Authors:** 


### Annotations.

### The Breeding of the Human.

An old saying tells us that the main duty of man is to plant trees and beget children. Both are necessary for posterity. But at the present day, at least in certain circles, the past claims no respect and the future gains no regard. Tree-growing and child-bearing are in danger of becoming lost arts. An urban environment has stunted man's interest in procreation. And yet now as ever, to those who interest themselves in the evolution of the race, no question is of more vital interest than that which concerns the breeding of the human. Mr. H. G. Wells, the brilliant romancist and essayist, has set himself the task of restating the question in the light of modern science. His studies on "Mankind in the Making," now appearing in the *Fortnightly Review* and the *Cosmopolitan*, are attracting much attention, and present much that is of the greatest interest. Every minute, he says, seven new citizens are added to the great English-speaking community which is scattered under various flags and governments throughout the world; so at all events, at present there is no immediate danger of the dying out of "English-speaking" babies. But the great question which is exercising thoughtful minds is—Are these the best possible babies? or, as Mr. Wells puts it, "Can we raise, and if so, what can we do to raise, the quality of the average birth?" The late Sir Douglas Galton suggested that "noble families" should collect "fine specimens of humanity" around them, with opportunities to devote their energies to the multiplication of a superior type. Superficial anthropologists and popular philanthropists have for long been enamoured of the idea of improving man by a process of artificial selection. Mr. Wells, we think, has well indicated the fallacy of such a proposition. He shows that we are, as a matter of fact, absolutely in the dark as to what points to breed for and what points to breed out. We have no definite standards. The suggested "points" are most variable—health, beauty, ability, capacity, genius, energy, and the like, concerning which we can never expect to arrive at a satisfactory quantitative definition. In fact ignorance, an absolute want of knowledge, stands in the way, and probably ever will, of securing with anything like precision the making of "a proper child."

### Operation Mania.

The introduction of anæsthetics, by aid of which the horrors of an operation are reduced to the mere discomfort of breathing a few times into a bag; the extended use of hospitals and nursing homes, which has the result of relieving relatives and friends of all the trouble and all the disagreeble incidents of an operation; and finally, the fact that with modern aseptic methods the scar left by it is often quite trivial, have conspired to make people regard the ordeal with curious indifference, and enter with a light heart into adventures from which they would perhaps have shrunk had they known a little more. And where operations are necessary all this is good. It has to be admitted, however, that there is another side to the question. Partly owing to the publicity given to operative work, partly to the fact that the successful case is apt to be by no means reticent about the advantages of "getting the thing over," and partly to the fact that dead men tell no tales, the public at large has come to look with unlimited and undue confidence upon operations as a way out of every difficulty—a *deus ex machinâ* which can always be invoked to hurry matters up should the treatment of a malady prove a little tedious. A curious sort of demand for operative treatment has arisen; people urge each other not to allow their doctors to "dally" with their cases, but to do something "radical"; and it is to be feared that sometimes if the doctor does not adopt this radical policy—or at least do something that requires an anæsthetic—they regard him as "old-fashioned" (the very hardest thing that one can nowadays say of any doctor), and run off to someone else. This is a kind of public sentiment which it is by no means easy to combat; the irresponsible chatter of the patient's friends condemns the cautious surgeon, while the unmeasured praise bestowed by the same irresponsible authorities upon the occasional success of an adventurous operator, leads to undeserved fame. As we need hardly say, the effect of all this must react injuriously upon the medical profession. Some medical men indeed assert that the evil consequences of this mania for operating have already attained considerable dimensions, and lead to much that is morally indefensible.

### Word-building by "Scientists."

Attention has lately been drawn to the extraordinary nomenclature in which scientific people are apt to indulge when christening new products. The *Practitioner* protests that new words must be invented to express new ideas, and that "the new wine of science cannot be poured into old verbal bottles without disastrous results." Nevertheless, the *Practitioner* is fain to admit that, especially in the department of pharmacology, there is much room for reform, adding in illustration of this opinion that in a German periodical which happened to be at hand, it was recorded that two learned pundits who tested the anæsthetic properties of acoine, known to chemists as "alkyloxyphenylguanidin," experimented with the following formidable sounding preparations:—

1. Triphenetylguanidinchlorhydrate.
2. Di-p-phenetyl-mono-anisylguanidinchlorhyde.
3. Triparaanisylguanidinchlorhydrate.
4. Diparaanisylmonophenetylguanidinchlorhydrate.
5. Diphenylmonophenetylguanidin.
6. Di-p-phenetyl-mono-ortho-phenetylguanidin.
7. Di-phenetyl-mono-phenylguanidinchlorhydrate.
8. Di-p-phenetyl-mono-ortho-anisylguanidinchlorhyde.
9. Di-p-phenetyl-mono-p-tolylguanidinchlorhyde.
10. Di-p-tolyl-mono-p-phenetylguanidinchlorhyde.

Such names, no doubt, are very useful as expressing in a condensed form the theory held at the moment as to the intimate chemical constitution of these substances. They have, however, this disadvantage that if the theory should change so must the name. In practice we believe that whenever these curious materials come into common use they are given more familiar names, often mere "slang" and meaningless terms, by which they are known for ordinary colloquial purposes. Of such are the whole tribe of "proprietary" appellations which figure so largely in the druggists' advertisements.

